# “Direct to Drug” screening as a precision medicine tool in multiple myeloma

**DOI:** 10.1038/s41408-020-0320-7

**Published:** 2020-05-11

**Authors:** Cecilia Bonolo de Campos, Nathalie Meurice, Joachim L. Petit, Alysia N. Polito, Yuan Xiao Zhu, Panwen Wang, Laura A. Bruins, Xuewei Wang, Ilsel D. Lopez Armenta, Susie A. Darvish, Greg J. Ahmann, Kimberly J. Henderson, Shulan Tian, Jonas J. Kruse, William M. Stewart, Jeremy T. Larsen, Craig B. Reeder, David Dingli, Prashant Kapoor, Shaji K. Kumar, Rafael Fonseca, P. Leif Bergsagel, Esteban Braggio, A. Keith Stewart

**Affiliations:** 1 Department of Hematology/Oncology, Mayo Clinic, Scottsdale, AZ USA; 2 Department of Department of Health Sciences Research, Mayo Clinic, Scottsdale, AZ USA; 3 Department of Department of Health Sciences Research, Mayo Clinic, Rochester, MN USA; 4 Department of Hematology/Oncology, Mayo Clinic, Rochester, MN USA; 5 Center for Individualized Medicine, Mayo Clinic, Rochester, MN USA

**Keywords:** Myeloma, Cancer therapy

## Abstract

Seventy-six FDA-approved oncology drugs and emerging therapeutics were evaluated in 25 multiple myeloma (MM) and 15 non-Hodgkin’s lymphoma cell lines and in 113 primary MM samples. Ex vivo drug sensitivities were mined for associations with clinical phenotype, cytogenetic, genetic mutation, and transcriptional profiles. In primary MM samples, proteasome inhibitors, dinaciclib, selinexor, venetoclax, auranofin, and histone deacetylating agents had the broadest cytotoxicity. Of interest, newly diagnosed patient samples were globally less sensitive especially to bromodomain inhibitors, inhibitors of receptor tyrosine kinases or non-receptor kinases, and DNA synthesis inhibitors. Clustering demonstrated six broad groupings of drug sensitivity linked with genomic biomarkers and clinical outcomes. For example, our findings mimic clinical observations of increased venetoclax responsiveness in t(11;14) patients but also identify an increased sensitivity profile in untreated patients, standard genetic risk, low plasma cell S-Phase, and in the absence of Gain(1q) and t(4;14). In contrast, increased ex vivo responsiveness to selinexor was associated with biomarkers of poor prognosis and later relapse patients. This “direct to drug” screening resource, paired with functional genomics, has the potential to successfully direct appropriate individualized therapeutic approaches in MM and to enrich clinical trials for likely responders.

## Introduction

Although a combination of immunomodulatory drugs (IMiDs) and proteasome inhibitors (PIs) is the current gold standard therapy for multiple myeloma (MM) with response rates in ~90% of newly diagnosed patients, deep complete remission is only achieved in ~50%, and most patients ultimately relapse, due to innate and acquired drug resistance^1–8^. Two challenges immediately become evident. Most urgent is the need to find alternatives for patients in whom these potent drug classes eventually fail^1,2,9–13^. Second, is to understand the mechanisms of resistance and to seek methodologies, dosing strategies, and new drug combinations that can prevent or overcome relapse^14–17^.

Because MM evolves secondary to acquired genetic events^18^, efforts toward individualizing treatments have, until now, focused on sequencing strategies^19–21^. Such early efforts have, however, failed to stimulate an era of precision medicine since actionable mutations are rare, subclonal, and lack matched therapeutics. Even when actionable mutations are present and the appropriate drug is utilized (e.g., treating *BRAF*^V600E^), clinical responses have been incomplete and transient^22,23^.

Therapy selection based on a demonstration of ex vivo individual sensitivity to a drug or drug combination offers a novel, attractive approach to individualized cancer therapy and is increasingly being explored in various cancers^24–31^. This is particularly relevant in MM given the large number of therapeutic agents currently available, either approved or under investigation, and the non-curable nature of the disorder. In that light, advances in functional drug screening technology and the rapid expansion of US Food and Drug Administration (FDA) approved oncology agents have now raised the prospect of a “direct to drug” screening approach to tailor therapies for MM. We therefore assembled a standardized MM drug panel (MMDP) and screening platform for drug profiling in both cell line models and primary samples. Baseline clinical phenotype, cytogenetics, DNA mutational profiles and RNA expression were recorded for each patient. This compendium provides a data-rich asset from which we explored clinical and genomic correlates of drug sensitivity. We identified subpopulations of patients with distinct drug sensitivity patterns linked to genetic and mutational profiles, and to clinical outcomes. These patterns illuminate vulnerabilities that can be exploited for future mechanism of action studies and combination therapy development.

## Materials and methods

### MM drug panel

The compounds of the MMDP were sourced from the NIH developmental therapeutics program and from various commercial vendors (Supplemental Table 1), dissolved in 100% dimethyl sulfoxide (DMSO) at a 10 mM stock concentration.

### Cell culture

All cell lines were provided by Dr. P. Leif Bergsagel’s laboratory and were fingerprinted to confirm their identity and tested negative for mycoplasma. The human myeloma cell lines (HMCLs) were maintained in RPMI-1640 supplemented with 5% FBS, and 1% pen/strep except for XG2, which was supplemented with 10% FBS and 1% pen/strep. For the non-Hodgkin’s lymphoma cell lines (NHLCLs), mantle and T-cell lymphoma subtypes were maintained in RPMI-1640 supplemented with 10% FBS and 1% pen/strep, while diffuse large B-cell lymphoma (DLBCL) subtypes were maintained in IMDM media supplemented with 20% human serum and 1% pen/strep.

### Human samples

Primary human MM cells were recovered from bone marrow aspirates collected from Mayo Clinic sites. Informed consent was given in writing for collection and research use under Institutional Review Board approval (IRBs 919-04, 521-93, 15-009436, 18-003198, 2207-02) in accordance with the Declaration of Helsinki. CD138 + cells were isolated by immunomagnetic bead selection (RoboSep; Stemcell Technologies). A minimum viable cell content of 500,000 cells and purity of 90% were required for drug screening on the same day. Baseline clinical characteristics were abstracted^32^, cytogenetics recorded from fluorescence in situ hybridization reports, and risk groups defined based on the mSMART 3.0 classification^2^.

### Drug sensitivity profiling platform

Feasibility studies for determination of assay time points and cellular density ranges were conducted in JJN3 cells by monitoring the efficacy of dinaciclib in real time every 2 h over 72 h using a nonlytic cellular viability assay (Real Time Glo, Promega). A lytic cellular viability assay (Cell Titer Glo, Promega) was used for all other experiments. For all cells amenable to culture (*n* = 40, Supplemental Table 2), cell densities were titrated and optimal seeding densities within linear ranges of luminescent signal were established at 24 hour (h) and 72 h drug exposure, per standard NIH assay guideline optimization criteria and methods. At time of experiment, 30 µL of cells suspension were plated onto assay-ready plates at optimal or consensus density (2000–3000 primary cells per well). In cell lines, the entire panel was tested at 24 h and 72 h drug exposure in duplicate as 7-point log dilutions, covering an assay concentration range of 0.01 nM–10 µM. In primary patient samples, cells were dispensed in assay titer plate wells covering the same assay concentration range, following the priority list of the MMDP until cells are depleted, and incubated for 24 h. Internal plate controls (positive control: wells treated with 10 µM staurosporine; negative control: 0.1% DMSO) were used for background subtraction and data normalization.

Since the screening assay was designed to potentially evolve as a clinically validated assay, no stromal layer was added in these initial analyses given the standardization difficulties. A 24 h readout was also preferred and validated given the known rapid demise of many primary MM cells in culture.

### Drug sensitivity data analysis

Dose-response curves were fitted in TIBCO Spotfire v7.0.0 after outlier removal, and curve maxima fixed to 100%. Curves were characterized by activity metrics including maximum response and half-maximal effective concentration. A compound was considered active following an inhibition of cellular viability greater than 20% for any given sample. The normalized area under the curve (AUC) was adopted as a unique metric for drug sensitivity. AUCs were calculated using the trapezoidal method implemented in GraphPad Prism v7.0.5 and normalized to negative controls. The normality of drug AUC distributions was tested using D’Agostino-Pearson omnibus method (GraphPad Prism v8). Differential sensitivities were evaluated based on the AUCs among classes of interest using the Mann–Whitney (2 variables) and Kruskal–Wallis (3 variables) tests as implemented in GraphPad PRISM v7.0.5 and TIBCO Spotfire v7.0.0. All *p*-values were not adjusted for multiple comparisons. Hierarchical clustering was performed using R package *ComplexHeatmap* (v1.99.5)^33^.

### Mutation and gene-expression profiling

Total RNA and DNA from the primary patient samples were isolated using the AllPrep DNA/RNA Kit (Qiagen #80204). We sequenced the entire coding regions of 139 genes using a customized 2.3 Mb SureSelect gene panel (M^3^P), covering 139 genes recurrently mutated, belonging to relevant pathways, consisting of actionable targets, or belonging to pathways targeted by the most commonly used drugs (PIs, IMiDs, and corticosteroids) in MM (Supplemental Table 3)^34–37^. Samples were paired-end sequenced (150 bp reads), using Illumina HiSeq 4000 sequencer with 24 samples assigned per lane of flow cell. The average coverage depth was >1000X per nucleotide, allowing the detection of mutations with variant allelic reads (VAR) as low as 1%. Raw variants were annotated using GATK variant annotator for variant quality^38^, somatic mutations were called using MuTect2 in tumor-only mode^39^, and Biological Reference Repository (BioR)^40^ for variant annotation with allele frequency available in public databases and for variant deleteriousness prediction. To remove germline mutations, common variants were eliminated based on the minor allele frequencies (>0.01%) available in one of the following germline variant databases: 1000 Genomes Project, ExAC and ESP6500, unless present in known MM mutation hotspots or in COSMIC. Additionally, we filtered out all variants with less than 10 supportive reads or found in less than 1% VAR.

A RNA-seq analysis workflow (MAP-RSeq^41^, v.3.0.1) was internally developed and used to perform a comprehensive analysis of raw RNA sequencing paired-end reads, which were aligned using a fast and splice-aware aligner (STAR^42^, v.2.5.2b) to the human genome build hg38. Quality control analysis was performed with RSeQC^43^ (v.3.0.0). Raw gene counts were quantified with FeatureCounts^44^ from the Subread package (http://subread.sourceforge.net/, v.1.5.1) and Transcripts Per Kilobase Million (TPM) were calculated.

## Results

### Creation of a phase 0 drug screening platform

A “direct to drug” strategy for drug sensitivity profiling was developed with a panel of 76 pre-screened small molecules comprising FDA-approved, cancer clinical trial, or biologically relevant emerging therapeutics. Since primary MM cell numbers can be limiting, compounds were rank-ordered for screening priority by likelihood of being clinically useful. The sensitivity of this MMDP was first profiled in a panel of 25 HMCLs (Supplemental Table 4) and then in a population of 113 primary myeloma patient samples (Supplemental Table 5). MM specificity was assessed in 15 NHLCLs (Supplemental Table 4). The baseline clinical, cytogenetic, and mutational profiles of the patient cohort were collected (Table 1).

**Table 1:** Summary of clinical and cytogenetic characteristics for the patient cohort.

Clinical/cytogenetic category	Total number of evaluable samples	Classes	Number of samples per class (%)
Diagnosis	113	Multiple myeloma (MM)	99 (87.6%)
		Smoldering MM (SMM)	14 (12.4%)
Disease status	113	Untreated	49 (43.4%)
		Early relapse	16 (14.1%)
		Later relapse	48 (42.5%)
Risk group (mSMART 3.0)	96 (MM)	Standard-risk	36 (37.5%)
		High-risk (HR)	60 (62.5%)
	60 (HR MM)	Double hit HR myeloma	17 (28.3%)
		Triple hit HR myeloma	5 (8.3%)
Cytogenetics—karyotype	104	Diploid	38 (36.5%)
		Hyperdiploid	61 (58.7%)
		Hypodiploid	5 (4.8%)
Cytogenetics—aberrations	110	t(11;14)/t(6;14)	30 (27.3%)
	110	Trisomies	57 (51.8%)
	110	t(4;14)	11 (10.0%)
	110	Del 17p	10 (9.1%)
	110	Gain(1q)	17 (15.5%)
	110	Del 13q	38 (34.5%)
	110	Monosomy 13	47 (42.7%)
	110	MYC aberration	17 (15.5%)

**Fig. 1:**
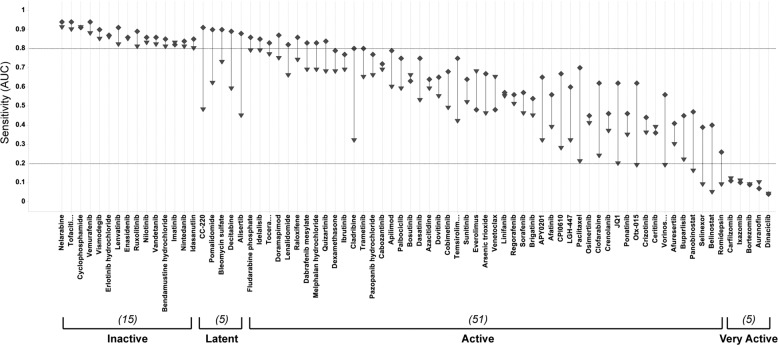
Validation of the 24 h time point in HMCLs. Plot of the sensitivity per drug at 24 h and 72 h drug incubation time points. Sensitivity is captured by the median AUC per drug, per time point (diamond for 24 h, triangle for 72 h). Drugs are rank-ordered by increasing sensitivity from left to right, and activity classes annotated under the plot. Counts of the number of drugs per class are indicated in parentheses. Except for the latent class, early detection of drug response at 24 h is found for 71/76 drugs in the panel (93%).

A feasibility study determined that dose-dependent efficacy could be reliably detected at 24 h with early detection of subsequent drug response correctly predicted in 93% of the panel (Fig. 1). In HMCLs, activities of only five drugs (7% of the MMDP), including IMiDs (pomalidomide and iberdomide), bleomycin sulfate, decitabine, and alisertib, were latent at 24 h and emerged only at 72 h. All other drugs demonstrably active at 72 h had discernable activity at 24 h. Fifteen drugs were inactive in HMCLs at both 24 h and 72 h, including the negative control, unmetabolized cyclophosphamide. Drug profiling results presented herein are for the 24 h incubation time point unless specified.

### Specificity of the MM screening panel

**Fig. 2:**
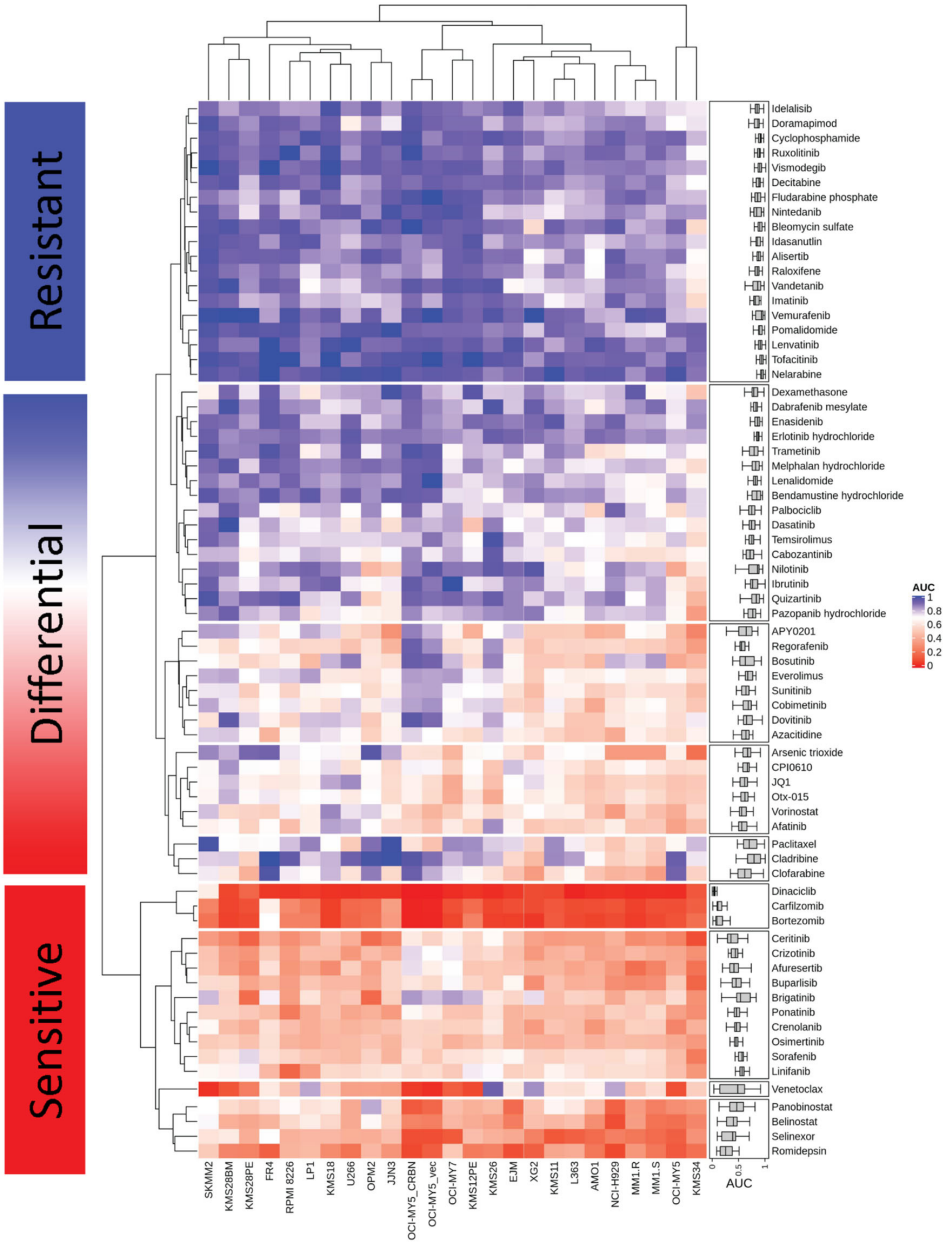
Heatmap of the drug sensitivities by unsupervised hierarchical clustering for the 25 HMCLs. The top section (resistant) includes the drugs that are mostly inactive in HMCLs. These include decitabine, tofacitinib, vismodegib, vemurafenib, idasanutlin, nelarabine, lenvatinib, fludarabine, and ruxolitinib. The bottom section (sensitive) includes broadly cytotoxic and very potent drugs in HMCLs, including proteasome inhibitors, dinaciclib, venetoclax, HDAC inhibitors, selinexor, ALK inhibitors, and some RTK inhibitors. The center area (differential) includes rapalogues, bromodomain inhibitors, MAPK inhibitors, some inhibitors of RTKs including FLT3, and PIKfyve inhibitors.

In HMCLs, the PIs (bortezomib, ixazomib, and carfilzomib), cyclin-dependent kinase inhibitor (dinaciclib), exportin 1 inhibitor (selinexor), the redox inhibitor auranofin, and histone deacetylating agents (HDACs: romidepsin, panobinostat) had the broadest cytotoxicity. Drug classes with more heterogeneous, but often deep, sensitivities in individual samples included bromodomain inhibitors, DNA synthesis inhibitors and kinase inhibitors (KIs), as captured on the HMCL-sensitivity map (Fig. 2). Among the differentially sensitive drugs, we noted novel MM target classes, notably PIKfyve inhibitors, which were active in over 90% of HMCLs. Data specifically focused on PIKfyve inhibition in hematological malignancies has been subsequently published^45–47^.

**Fig. 3:**
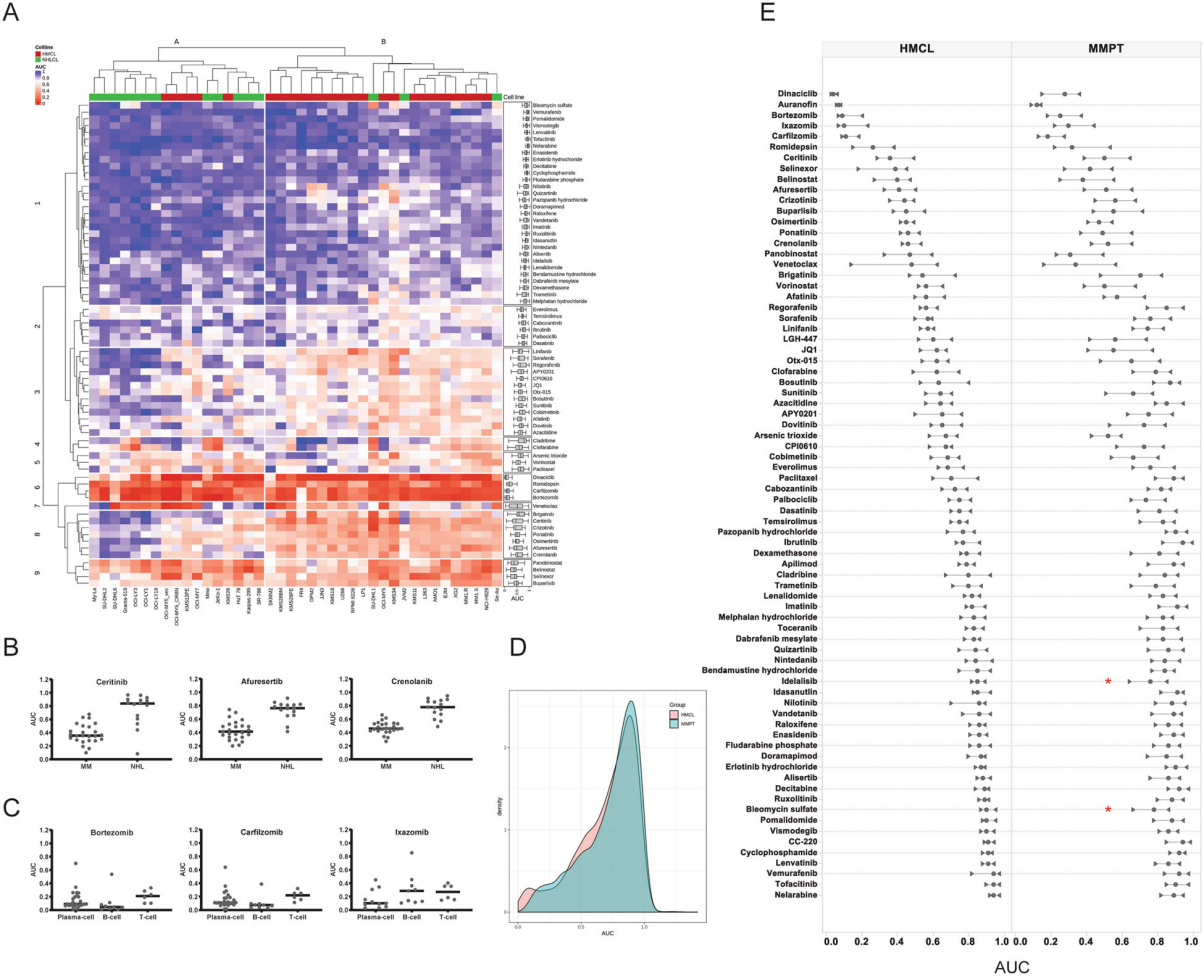
Drug sensitivity profiling in HMCLs and NHLCLs highlights MM specificity of the MMDP and high similarity of drug sensitivity landscapes in HMCLs and in MM primary samples. **a** Hierarchical clustering analysis of drug sensitivities (AUCs) identifies two dominant cell line clusters (column clusters A and B) defining seven major drug subgroups (row clusters 1–7). Individual drug sensitivity distributions are inserted as box plots on the right side of the heatmap. The heatmap shows that for most drug classes except the broadly cytotoxic ones (row clusters 6 and 9), the MMDP drugs are overall more sensitive in MM than in NHL. **b** Scatter plot of the drug sensitivity (AUC) by cell line subgroup; horizontal bars represent the median AUC. The ALK inhibitor ceritinib, the AKT inhibitor afuresertib, and the PDGFR/FLT3 inhibitor crenolanib are highly sensitive in HMCLs while they are poorly sensitive in NHLCLs. The dataset of all drugs with significant differential sensitivity in HMCLs *versus* NHLCLs is provided in Supplemental Table 6. **c** Scatter plot of the drug sensitivity (AUC) by cell type; horizontal bars represent the median AUC. The PIs bortezomib and carfilzomib were more sensitive in plasma cell (HMCLs) than in T-cell NHLCLs but less than in B-cell NHLCLs; Ixazomib was more specific to MM overall. **d** AUC density plots using Gaussian kernel for HMCLs (red) and 99 primary samples in our cohort with MM diagnosis (MMPT, cyan). AUCs fall in the 0~1.0 interval for the most, and distributions are consistent with each other for both sample types, which may imply that the drugs perform similarly on myeloma primary patient samples as they do on the cell lines. **e** Comparative plot of the interquartile range per drug in HMCLs (left) and MMPTs (right); circles represent the median AUC for each drug, while right and left triangles indicate the value of their first and third quartiles, respectively. Drugs are rank-ordered from top to bottom by increasing median AUC value in HMCLs, i.e., by decreasing sensitivity. The ex vivo sensitivity by drug follows the trends observed in vitro, with interquartile ranges slightly broader in primary samples. While this can partly originate from a higher intrinsic ex vivo assay noise and higher heterogeneity of primary samples as compared to cell lines, this can also importantly pinpoint shifts in differential responses associated with cytogenetic aberrations and occurrence of mutations. Two such cases are highlighted on the plot with red stars for idelalisib and bleomycin, for which our statistical analysis demonstrated that sensitivity of primary patients to these drugs is higher in presence of *IDH* mutations.

To evaluate specificity of the MMDP for MM *versus* other hematological malignancies, the panel was counter-screened in 15 NHLCLs. The chemosensitivities of drugs tested across all 40 cell lines were analyzed using unsupervised hierarchical clustering (UHC). Two dominant groups were distinguished by NHLCLs and HMCLs, respectively (Fig. 3a). Thirty-three agents (43% MMDP) had AUCs >5% lower in HMCLs than in NHLCLs, indicating an increased sensitivity in MM. Differential response analysis between MM and NHL confirmed statistical significance for 26 of these compounds. Of these, 18 were kinase inhibitors targeting MAPK, PDGFRs, EGFR, ALK, FLT3, AKT, and CDKs (Fig. 3b, Supplemental Table 6). PIs were more active in MM and B-cell NHLCLs than in T-cell NHLCLs (Fig. 3c). The BCL-2 inhibitor venetoclax was more sensitive in HMCLs and B-cell NHL than in T-cell NHL. Nine agents were more responsive in NHLCLs than HMCLs (significant for five). These included three HDAC inhibitors (vorinostat, panobinostat, and romidepsin) that were highly efficacious in both NHL groups, yet more sensitive in T-cell NHLCLs.

### The landscape of patient-derived MM cell drug sensitivity

The MMDP was then profiled at 24 h exposure in 113 primary patient samples. Drugs were screened ex vivo by MMDP priority order until cells were depleted, and an average of 58 drugs (76% MMDP) was tested per sample. The AUC distributions, both global and at the individual drug levels, were more similar for cell lines in vitro and primary samples ex vivo than we would have initially predicted (Fig. 3d, e). Consistent with the HMCLs, 14 drugs were active in <10% of primary samples at 24 h. These included iberdomide, ibrutinib, decitabine, erlotinib, tofacitinib, vismodegib, idasanutlin, vemurafenib, nelarabine, lenvatinib, paclitaxel, nintedanib, enasidenib, and unmetabolized cyclophosphamide. The most potent drugs with >40% samples presenting with an EC_50_ below 100 nM were the three PIs (bortezomib, carfilzomib, and ixazomib), selinexor, venetoclax, panobinostat, romidepsin, dinaciclib, and auranofin. Eighteen drugs were active (albeit often at higher concentrations) in over 80% of primary samples tested, including the PIs, selinexor, venetoclax, HDAC inhibitors (panobinostat, romidepsin, belinostat), arsenic trioxide, auranofin, and eight KIs (dinaciclib, afuresertib, buparlisib, ceritinib, crizotinib, crenolanib, ponatinib, and osimertinib).

For PIs specifically, primary patient samples were more sensitive to carfilzomib (median EC_50_ 0.92 nM; median maximum response 85.76%) than to bortezomib and ixazomib (median EC_50_ 10.19 nM and 95.32 nM; median maximum response 79.89% and 79.00%, respectively). A deep response for carfilzomib, with EC_50_ < 10 nM and maximum response >80%, was observed for 72% of samples, a number that drops to 21% for bortezomib. Additionally, 51% of samples had partial bortezomib response (<80% inhibition), as compared to 17% for carfilzomib. Ixazomib was the least active PI with all EC_50_s > 100 nM, 13% of which were in micromolar range, as compared to 5% for bortezomib and none for carfilzomib. Another commonly employed agent, dexamethasone, was tested in 89 samples. Of these, 24 showed response with efficacies at <10 nM and variable response depths (% cellular inhibition) ranging from 20 to 60% (*n* = 18) to >60% (*n* = 6). Overall dexamethasone sensitivity distributions at 24 h exposure were similar in HMCLs and primary samples, thus our 24 h assay results collectively indicate that our platform could identify highly sensitive responders to dexamethasone.

### Phenotypic correlation with ex vivo sensitivity

Baseline profiles were abstracted, creating a compendium inclusive of clinical disease status, risk groups, and chromosomal aberrations for each patient (Supplemental Table 7). Mutations were captured for 73 primary samples in which DNA was available (Supplemental Table 8). Six patients lacked mutations in the studied genes, while 67 cases (92%) had an average of three mutations each. The most frequently mutated genes per sample were: *KRAS* (36%), *NRAS* (22%), *ATM* (18%), *ZFHX4* (14%), *TP53* (12%), *FAM46C* (12%), *DIS3* (11%), *BRAF* (10%), *IRF4* (7%), and *CUL4B* (5%).

**Fig. 4:**
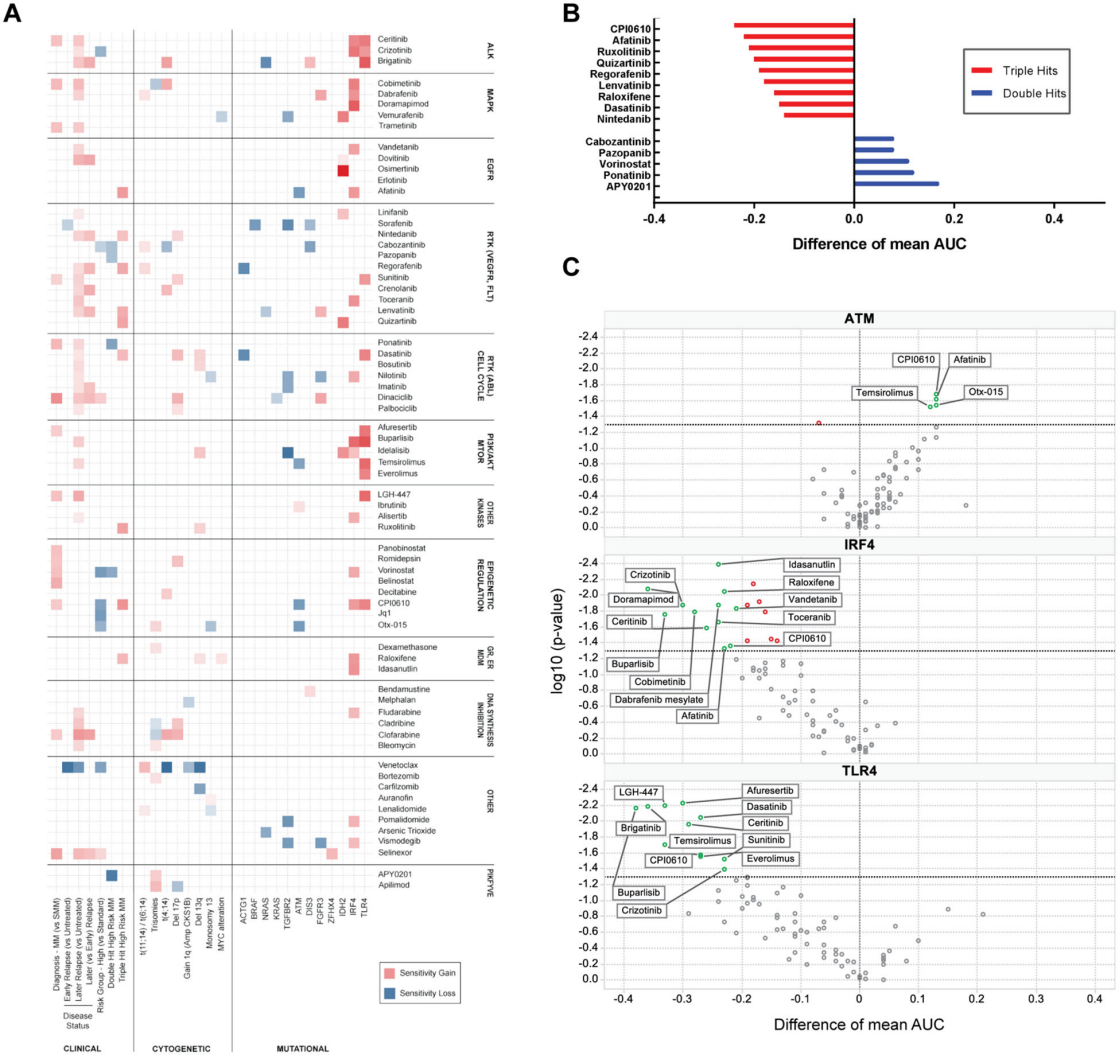
Clinical stratification, genetic aberrations, and mutational status modulate sensitivity to MMDP agents in MM. **a** Differential sensitivity heatmap of the statistically significant associations between drugs and clinical stratification, chromosomal aberration status, and mutational profiles. Red indicates gain of sensitivity in the class under consideration (*versus* reference or absence of aberration), while blue indicates a loss of sensitivity. The more intense the color, the larger the sensitivity difference. Data are reported for all differential activities with statistical significance (*p* < 0.05); numerical data and individual box plots are provided in Supplemental Table 9. Signal transduction inhibitors targeting ALK, MAPK, PI3K/AKT, CDKs, and RTKs, as well as HDAC inhibitors and selinexor, had enhanced sensitivity in MM as compared to SMM. KIs across all classes, DNA synthesis inhibitors, and selinexor were more sensitive in later relapse MM samples while venetoclax was more sensitive in untreated samples. Venetoclax, crizotinib, cabozantinib, vorinostat, and bromodomain inhibitors were less responsive in high-risk MM than in standard risk, while selinexor and dinaciclib had increased sensitivity for high-risk samples. **b** Histogram of differential drug sensitivity (difference of mean AUC between present/absent classes) for double hit high-risk samples (blue) and triple hit high-risk samples (red). Positive values indicate a lower sensitivity while negative values reflect a higher sensitivity in the hit class as compared to high risk (no hit). Double hit high risk is generally associated with drug resistance while triple hit high risk favors drug sensitivity. **c** Volcano plot of the differential drug sensitivity (difference of mean AUC between present/absent classes) for *ATM*, *IRF4*, and *TLR4* genes. Positive values indicate a lower sensitivity, while negative values reflect a higher sensitivity in the mutated class as compared to non-mutated class. Drugs with *p* < 0.05 are labeled on the plot. Mutations in *ATM* were overall detrimental to sensitivity while mutations in *IRF4*, and *TLR4* were overall favorable to sensitivity. A loss of sensitivity of BET inhibitors CPI0610 and otx-015 was observed in presence of mutations of DNA damage repair associated *ATM* gene. Inversely, mutations of *IRF4* and *TLR4* genes associated with a broad increase in sensitivity across multiple drug classes involving signal transduction through receptor and non-receptor kinases. Indeed, samples harboring *IRF4* mutations, which have previously been linked to better prognosis were exquisitely sensitive to ALK inhibitors ceritinib and crizotinib as well as 3 out of the 5 tested MAPK inhibitors (cobimetinib, dabrafenib, and doramapimod). When compared to non-mutated samples, samples with *TLR4* mutations had better sensitivity to all three tested ALK inhibitors as well as 4 out of 5 PI3K/mTOR inhibitors.

For each panel drug, a global analysis of differential drug sensitivity demonstrated significant patterns of enhanced or reduced sensitivity linked to clinical groups, and presence/absence of chromosomal aberrations and mutations (Fig. 4a, Supplemental Table 9).

First, we noted that active MM samples were significantly more sensitive than smoldering MM (SMM) samples to all studied HDAC inhibitors, selinexor, the signal transduction inhibitors targeting ALK (ceritinib), MAPK (cobimetinib, trametinib), CDKs (dinaciclib), RTKs (sunitinib, ponatinib), and AKT (afuresertib).

Second, we determined that later relapse MM samples, defined as >2 relapses, appeared more sensitive than untreated disease to kinase inhibition across all classes represented in the panel (26/40 inhibitors), to selinexor and four of the DNA synthesis inhibitors. Venetoclax and bromodomain inhibitors were more potent in standard genetic risk MM, while selinexor was more potent in high genetic risk MM samples.

Finally, differential drug response across cytogenetic classes revealed significant sensitivity changes based on the presence of genetic aberrations, including higher sensitivity of venetoclax in t(11;14), described in detail below. CDK inhibitors (dinaciclib and palbociclib) and DNA synthesis inhibitors (cladribine and clofarabine) had stronger efficacy in samples with 17p deletion^48^. Double hit MM samples had lower sensitivity than single hit, high-risk samples to cabozantinib, pazopanib, ponatinib, and vorinostat, while triple hit MM samples appeared exquisitely sensitive to seven KIs, CPI0610, and raloxifene (Fig. 4b).

We also examined the associations between drug sensitivity and mutational profiles. For statistical significance, our analysis was restricted to the 25 genes with mutations in three or more patient samples. Mutations of *ACTG1*, *BRAF*, *NRAS*, *KRAS*, *TGFBR2*, and *ATM* genes appeared detrimental to overall sensitivity while mutations of *IDH2*, *IRF4*, and *TLR4* led to favorable chemosensitivity changes (Fig. 4c), as compared to the non-mutated samples in each class. Notably *IRF4* mutation has previously been associated with good clinical outcomes, an observation that lends credence to our findings.

### Drug sensitivity confirms venetoclax contextual sensitivity

As a “proof of concept” validation of the panel’s potential clinical utility, cellular efficacy of single agent venetoclax was first measured in 25 HMCLs. After 24 h drug incubation, 92% of HMCLs demonstrated dose-dependent response to the drug, with mid-point EC_50_ ranging from <0.1 to >10000 nM and AUCs varying widely from 0.03214 to 0.9187. Twenty-two HMCLs had known IgH translocations, four of which harbored a t(11;14). Significant differential activity profile was demonstrated between HMCLs harboring t(11;14) (4 HMCLs; median AUC 0.1153) and lacking t(11;14) (18 HMCLs; median AUC 0.5499) (*p* = 0.0074).

**Fig. 5:**
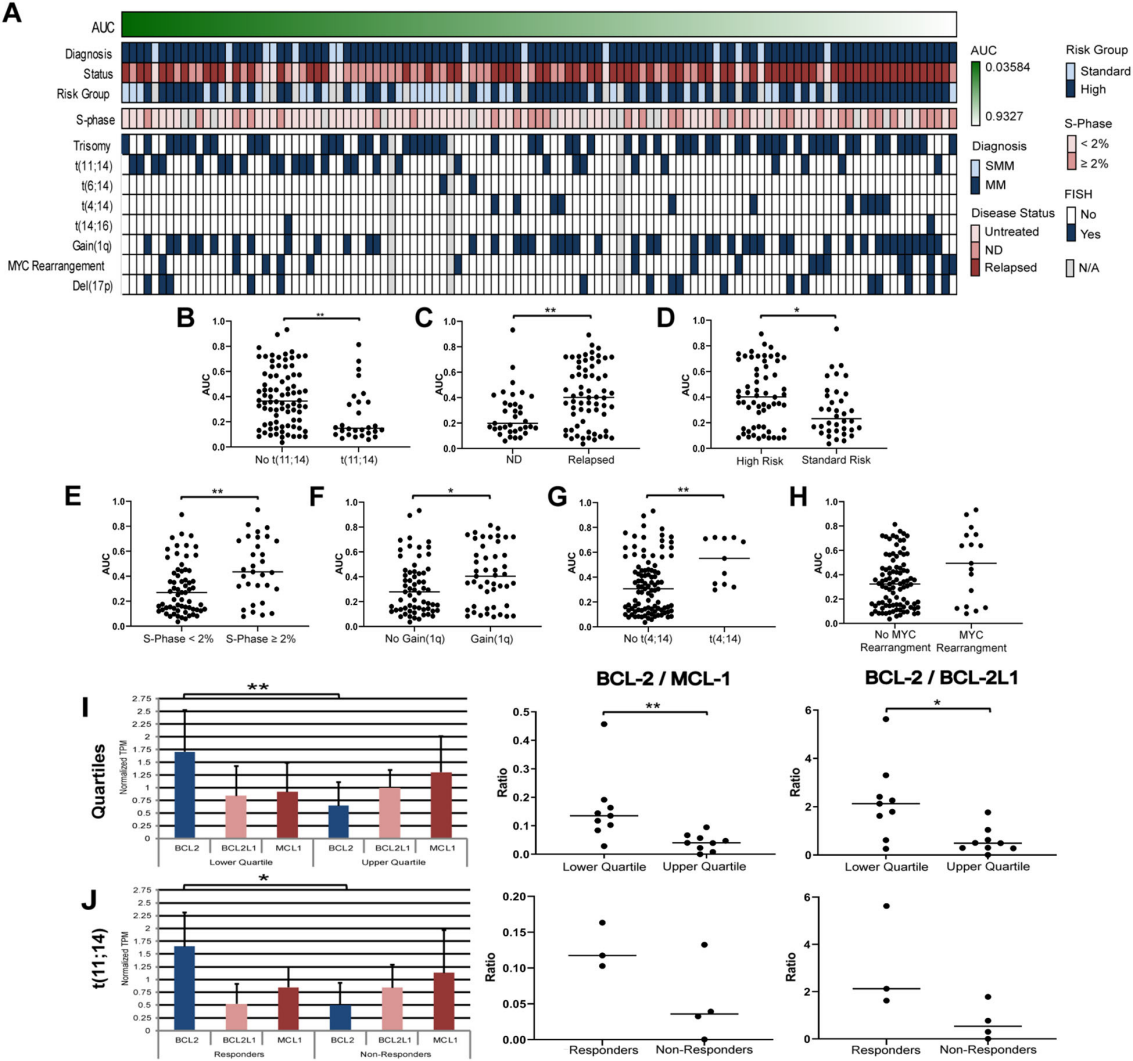
Venetoclax sensitivity in 113 MM ex vivo primary patient samples. **a** Heatmap showing cellular efficacy of single agent venetoclax response following a 24 h drug exposure in 113 primary patient samples rank-ordered by drug sensitivity, associated to clinical data including diagnosis, MM disease status, mSMART 3.0 risk group classification for active MM, flow cytometry S-Phase analysis, and FISH cytogenetics. Missing data are colored in gray. **b–h** Plots of venetoclax differential sensitivity by class showing: **b** increased ex vivo sensitivity of venetoclax in presence of t(11;14) (*n* = 28; median AUC 0.1491) when compared to samples lacking the translocation (*n* = 82; median AUC 0.3644) (Mann–Whitney test; *p* = 0.0013); **c** increased ex vivo sensitivity to venetoclax associated to newly diagnosed MM (*n* = 35; median AUC 0.1977) when compared to relapsed MM (*n* = 64; median AUC 0.4025) (Mann–Whitney test; *p* = 0.0041); **d** increased ex vivo sensitivity to venetoclax associated to standard risk (*n* = 36; median AUC 0.2324) when compared to high risk (*n* = 60; median AUC 0.4025) (Mann–Whitney test; *p* = 0.0199); **e** increased ex vivo sensitivity to venetoclax associated to low plasma cell S-Phase (*n* = 65; median AUC 0.2697) when compared to high S-Phase (*n* = 31; median AUC 0.4353) (Mann–Whitney test; *p* = 0.0035); **f** increased ex vivo sensitivity to venetoclax associated to samples lacking Gain(1q) (*n* = 63; median AUC 0.2788) when compared to samples with Gain(1q) (*n* = 47; median AUC 0.4047) (Mann–Whitney test; *p* = 0.0383); **g** increased ex vivo sensitivity to venetoclax associated to samples lacking t(4;14) (*n* = 99; median AUC 0.3070) when compared to samples harboring the translocation (*n* = 11 samples; median AUC 0.5514) (Mann–Whitney test; *p* = 0.01); **h** a trend towards significance with MYC rearrangements associated to lower venetoclax sensitivity (*n* = 17; median AUC 0.4940) when compared to samples without rearrangements (*n* = 93; median AUC 0.3238) (Mann–Whitney test; *p* = 0.0658). **i–j** Transcriptomic ratios of anti-apoptotic BCL-2 family members, showing: **i** *BCL2* expression was significantly increased in the lower quartile (responders) when compared to the upper quartile (non-responders) (*t*-test, *p* = 0.0036), with a significant difference in *BCL2/MCL1* ratio (Mann–Whitney test; *p* = 0.0019) and *BCL2/BCL2L1* ratio (Mann–Whitney test; *p* = 0.0106) between the two groups. **j** Ex vivo venetoclax response distinguished responders and non-responders in samples harboring t(11;14). *BCL2* expression was also significantly increased in the t(11;14) responders (*t*-test, *p* = 0. 0.0379) when compared to the t(11;14) non-responders. Increased *BCL2/MCL1* and *BCL2/BCL2L1* ratios were associated with the responders; however, statistical significance was not reached likely due to the relative low number of samples in each subgroup.

Venetoclax sensitivity was then measured in 113 primary patient samples, 28 of whom harbored a t(11;14) and 64 of whom were relapsed patients that had received a median of three therapies (range 1–15) prior to the ex vivo drug screen. As seen with HMCLs, most samples (93%) exhibited a dose-dependent response to the drug; a broad mid-point EC_50_ range was observed (<0.1 to >10000 nM) and AUCs varied from 0.03584 to 0.9327. The global potential of venetoclax in MM was demonstrable with 49% of patient samples exhibiting efficacies <100 nM and only 7% of samples classified as completely inactive (Fig. 5a).

Clinical features, including disease status and cytogenetics, were associated with ex vivo venetoclax sensitivity (Fig. 5a). As expected, samples from patients harboring t(11;14) had increased ex vivo sensitivity to the drug when compared to patients lacking the translocation (Fig. 5b). The two samples from patients harboring t(6;14) (which also upregulates a cyclin protein) also demonstrated exquisite sensitivity to venetoclax, with mid-point EC_50_ of 47.1 nM and 50.5 nM and AUCs of 0.2806 and 0.2513, respectively.

Increased ex vivo sensitivity to venetoclax was demonstrated in newly diagnosed MM samples when compared to relapsed MM (Fig. 5c), and in standard risk when compared to high risk (Fig. 5d). Other cytogenetic characteristics associated with increased venetoclax sensitivity were low plasma cell S-Phase (Fig. 5e); samples lacking Gain(1q) (Fig. 5f); and samples lacking t(4;14) (Fig. 5g). Finally, there was a trend towards significance with MYC rearrangements associated with decreased venetoclax sensitivity (Fig. 5h).

In acute myelogenous leukemia (AML), *IDH1/2* mutations causing *BCL2* dependence related to venetoclax sensitivity has been reported^49^. In our study, three patients harboring *IDH* mutations exhibited profound venetoclax sensitivity, *i.e.*, one patient sample with an *IDH1* mutation had an AUC of 0.0979, and two patients with *IDH2* mutations had AUCs of 0.0358 and 0.3200. Finally, samples with *DIS3* mutations trended to negatively associate with venetoclax response (9 samples; AUC 0.4539) when compared to samples without mutation (63 samples; AUC 0.307) (*p* = 0.0628).

We next investigated the predictive value of anti-apoptotic *BCL2* family member transcriptomic ratios as biomarkers of venetoclax sensitivity. RNA-seq analysis was available in 38 of the 99 MM primary patient samples. We first selected the nine most (median AUC 0.09409) and least (median AUC 0.7195) sensitive samples to venetoclax. Overall *BCL2* expression was significantly increased in responders when compared to non-responders. In addition, non-responders had an increased *BCL2L1* and *MCL1* expression, leading to a significant difference in *BCL2*/*MCL1* and *BCL2*/*BCL2L1* ratios between the responder and non-responder groups (Fig. 5i).

Although the presence of t(11;14) was significantly associated with venetoclax sensitivity, there were outlier samples harboring t(11;14) that were poorly responsive (Fig. 5b). Therefore, we further evaluated the *BCL2* family transcriptomic ratios in seven t(11;14) samples. The responders had a significantly increased *BCL2* expression and decreased *BCL2L1* and *MCL1* expression when compared to non-responders, resulting in increased *BCL2*/*MCL1* and *BCL2*/*BCL2L1* ratios (Fig. 5j).

### Clinical outcomes highlight potential clinical utility

Two relapsed MM patients harboring t(11;14) and one hyperdiploid patient were treated with venetoclax immediately after the drug screen. The hyperdiploid patient was most interesting. This patient was in fifth relapse, with previous treatment protocols including lenalidomide, pomalidomide, bortezomib, carfilzomib, and daratumumab. Unexpectedly, given the lack of t(11;14), venetoclax was the most potent agent within the screen (AUC of 0.3277). The patient was treated with venetoclax, achieving a partial response that lasted for ten months. This case exemplifies the significant clinical impact that ex vivo drug screening can have for drug selection and clinical outcome, especially in advanced stage of disease with limited therapeutic options.

Although the ex vivo screen results in t(11;14) patients predicted only moderate sensitivity, with AUCs of 0.3380 and 0.3575 (at the 53rd and 57th percentile of venetoclax sensitivity distribution in the studied population) both had a partial response to combination therapy. One patient had relapsed for the second time and was treated with venetoclax and dexamethasone. The other had relapsed for the eighth time and was treated with the combination of venetoclax, carfilzomib, and dexamethasone, which was considered not evaluable for single agent response.

### Selinexor sensitivity is higher in poor prognosis patients

**Fig. 6:**
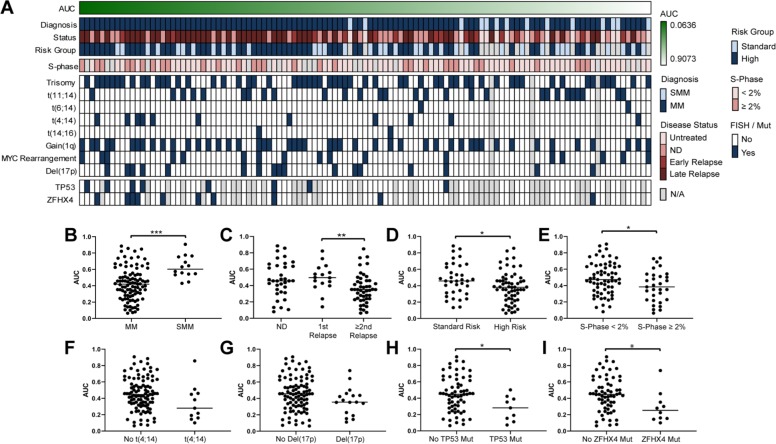
Selinexor sensitivity in 113 MM ex vivo primary patient samples. **a** Heatmap showing cellular efficacy of single agent selinexor response following a 24 h drug exposure in 113 primary patient samples rank-ordered by drug sensitivity, associated to clinical data including diagnosis, MM disease status, mSMART 3.0 risk group classification for active MM, flow cytometry S-Phase analysis, FISH cytogenetics, and *TP53* and *ZFHX4* mutation status. Missing data are colored in grey. **b–i** Plots of selinexor differential sensitivity by class showing: **b** increased ex vivo sensitivity to selinexor associated to samples from patients with MM (*n* = 99; median AUC 0.4198) when compared to patients with SMM (*n* = 14; median AUC 0.6029) (Mann–Whitney test; *p* < 0.0001); **c** a trend towards statistical significance of increased ex vivo sensitivity to selinexor associated to relapsed MM (1st and ≥2 relapsed groups combined; *n* = 64; median AUC 0.3844) when compared to newly diagnosed MM (*n* = 35; median AUC 0.4570) (Mann–Whitney test; *p* = 0.0647) and a significant increase in sensitivity in patients at a second or further relapse (*n* = 48; median AUC 0.3518) when compared to the first relapse (*n* = 16; median AUC 0.4974) (Mann–Whitney test; *p* = 0.0046); **d** increased ex vivo sensitivity to selinexor associated to high mSMART risk (*n* = 60; median AUC 0.3804) when compared to standard risk (*n* = 36; median AUC 0.4573) (Mann–Whitney test; *p* = 0.0289); **e** increased ex vivo sensitivity to selinexor associated to high plasma cell S-Phase (*n* = 31; median AUC 0.3840) when compared to low S-Phase (*n* = 65; median AUC 0.4738) (Mann–Whitney test; *p* = 0.0305); **f** a trend towards statistical significance of increased ex vivo sensitivity to selinexor associated to samples harboring t(4;14) (*n* = 11; median AUC 0.2796) when compared to samples lacking the translocation (*n* = 99; median AUC 0.4465) (Mann–Whitney test; *p* = 0.0676) or **g** in samples presenting a 17p deletion (*n* = 17; median AUC 0.3542) when compared to samples without the deletion (*n* = 93; median AUC 0.4559) (Mann–Whitney test; *p* = 0.0883); **h** increased ex vivo sensitivity to selinexor associated to samples with *TP53* mutations (*n* = 9; median AUC 0.2814) when compared to samples without the mutation (*n* = 64; median AUC 0.4480) (Mann–Whitney test; *p* = 0.0212); **i** increased ex vivo sensitivity to selinexor associated to *ZFHX4* mutations (*n* = 10; median AUC 0.2527) when compared to samples without the mutation (*n* = 63; median AUC 0.4475) (Mann–Whitney test; *p* = 0.0197).

In another deep dive to examine the recently FDA-approved drug selinexor, we explored additional potential predictive biomarkers of response to this drug. While 90% of the 113 primary patient samples exhibited dose-dependent response to selinexor, characterized by broad EC_50_ ranges (<1 to >10000 nM), 55% of samples had efficacies <100 nM, with poor prognosis clinical characteristics associated with increased selinexor response (Fig. 6a). SMM samples were less sensitive to selinexor when compared to MM samples (Fig. 6b), while later relapse MM samples were significantly more sensitive than first relapse samples (Fig. 6c). Increased sensitivity was also found in samples from high-risk patient samples (Fig. 6d) and high plasma cell S-Phase (Fig. 6e). We also noted a trend towards significance indicative of increased selinexor sensitivity in samples harboring t(4;14) (Fig. 6f) or 17p deletion (Fig. 6g). Finally, selinexor sensitivity was higher in samples with *TP53* or *ZFHX4* mutations (Fig. 6h, i), known to negatively impact MM^50^.

### Ex vivo profiling identifies patterns of drug sensitivity

**Fig. 7:**
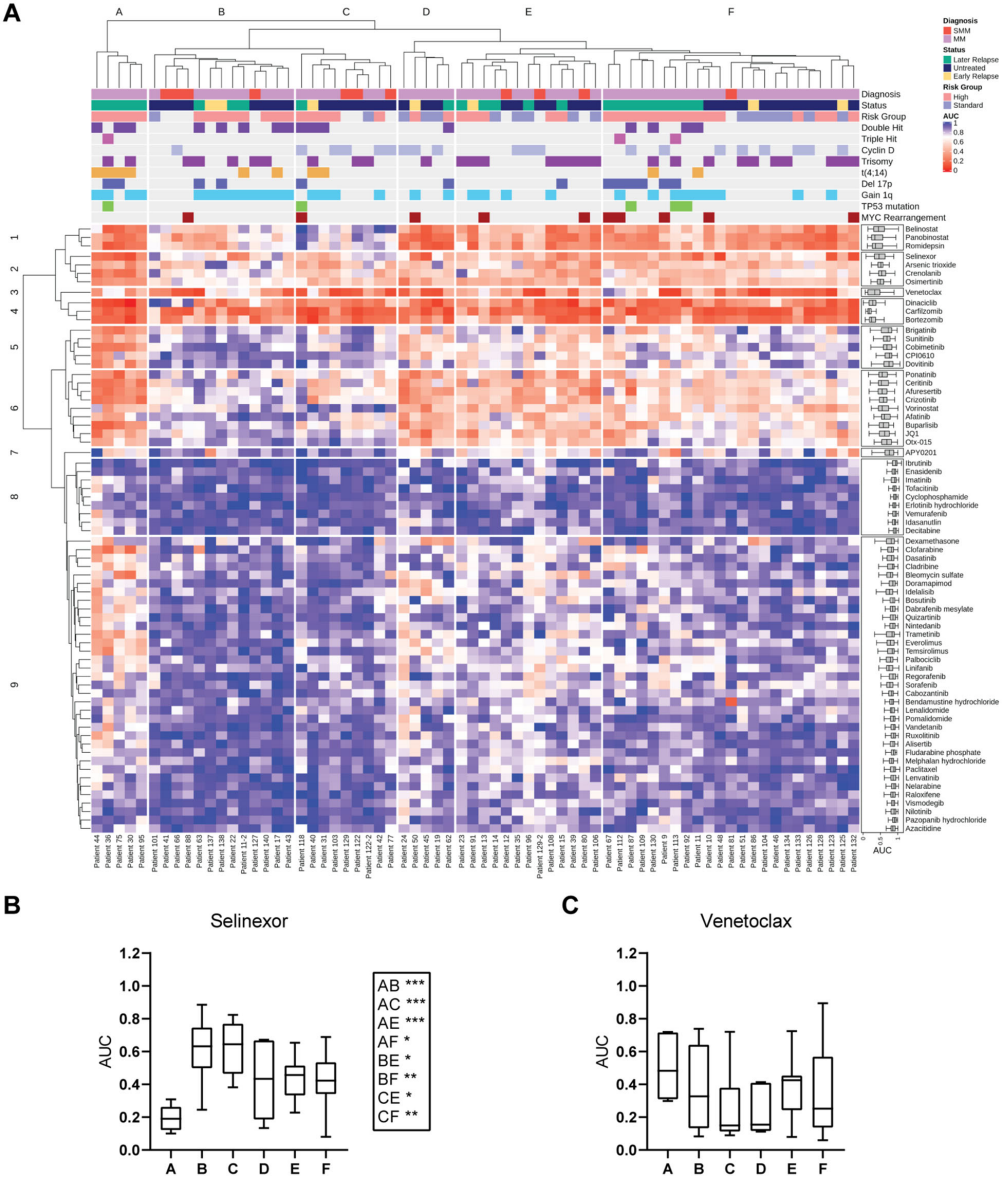
Ex vivo drug sensitivity profiles of 70 drugs in 68 patient samples define sample subgroups with differential chemosensitivity and illuminate contextual susceptibilities to MM drugs. **a** Unsupervised hierarchical clustering analysis of drug sensitivities measured by AUCs identified nine drug clusters (rows) defining six major patient clusters (columns, **A**–**F**). Group A was sensitive to the most drug classes (mean AUC(A) 0.5697). Groups D and E had intermediate sensitivity (mean AUC(D) 0.6409; mean AUC(E) 0.6761) with very similar chemosensitivity profiles, followed by group F (mean AUC(F) 0.7301). Clusters B and C were the most drug resistant (mean AUC(C) 0.7881; mean AUC(B) 0.8209). The clinical profile, cytogenetic classification, and mutational profile by sample group are annotated above the heatmap and summarized in Supplemental Table 10. Row cluster 1 is enriched in HDAC inhibitors. Row cluster 3 isolated venetoclax as a singleton, revealing the unique drug sensitivity profile of this drug. PIs were grouped in row cluster 4. Row clusters 2, 5, 6, and 9 contained kinase inhibitors targeting ALK, MAPK, EGFR, and PI3K/mTOR, which had enhanced sensitivity in group A as compared to resistant groups B and C. Group A was also more sensitive to DNA synthesis inhibitors and was enriched by later relapse MM samples characterized also by high risk genomic abnormalities including t(4;14) and/or gain of chromosome 1q. The most resistant groups B and C in contrast were enriched for untreated or early relapse samples with high risk stratification. They primarily differed from each other by t(11;14) status (Mann–Whitney test; *p* = 0.0132) and sensitivity to PIs. HDAC inhibitors and select KIs also had differential chemosensitivity profiles between these two resistant groups (Fig. 8). The three intermediate sensitivity groups (clusters D, E, and F) retained sensitivity to ALK inhibitors and BET inhibitors lost in resistant groups B and C (row cluster 6). (B-C) Box plots of the drug sensitivity (AUCs) by sample subgroup, showing: **b** Differential sensitivity of selinexor by sample group, with exquisite selinexor sensitivity of samples in cluster A as compared to resistant groups B and C, and to intermediate groups E and F; **c** Differential sensitivity of venetoclax by sample group, with group A trending as the least sensitive of all.

**Fig. 8:**
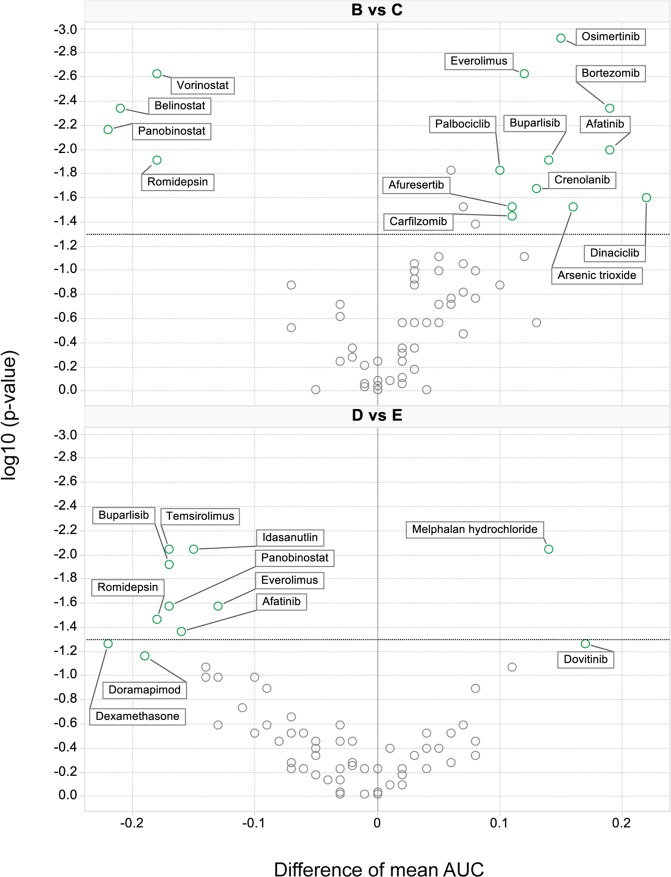
Volcano plots of the differential drug sensitivity between UHC groups. Volcano plots identify drugs with differential sensitivity between patient subpopulations (mean AUC difference between two classes). The upper panel identifies drugs, which are more sensitive in resistant group B than C (left) and inversely more sensitive in group C than B (right); the bottom panel provides the same analysis for groups D *versus* E. Drug annotation is provided in Supplemental Table 1. Group B has significantly higher sensitivity to HDAC inhibitors, while group C has higher sensitivity than B to agents inhibiting PI3K/mTOR, cell cycle, and the proteasome. Group D is more sensitive than E to dexamethasone, HDAC inhibitors, PI3K/mTOR inhibitors, and to agents targeting MAPK and EGFR, while group E is more sensitive than D to melphalan.

To further identify more subtle drug response patterns, an ex vivo data subset from 68 primary samples tested against 70 MMDP agents (without missing data) was examined by clustering analysis. As shown in Fig. 7a, six response groups were delineated (A–F) based on drug sensitivity profile similarities across patient samples. Differential chemosensitivity analysis by groups showed that samples in group A had exquisite sensitivity to selinexor (Fig. 7b) while concomitantly having the lowest sensitivity to venetoclax (Fig. 7c). This patient subpopulation was enriched with later relapse MM samples, with high-risk genomic abnormalities including t(4;14) (*n* = 4) and/or Gain(1q) (*n* = 4), *i.e.*, poor prognosis factors which we had associated with increased selinexor sensitivity but were detrimental to venetoclax sensitivity. Differential chemosensitivity profiles are further shown in Fig. 8. These promising results suggest that as our sample numbers increase and additional multi-omics are integrated, subpopulations of outstanding or poor responders and their contextual vulnerabilities could be isolated by this platform.

## Discussion

Advances in screening technology and clinical availability of a large arsenal of oncology drugs have enabled “direct to drug” screening as a route to individualized therapy for MM. To this end, we have assembled a comprehensive database of in vitro and ex vivo sensitivities in cell lines and patient primary cells each exposed to the MMDP, a panel of select oncology drugs with MM significance. Additionally, for each patient sample, a baseline clinical dataset was collected, incorporating clinical demographics, genetic subtype, disease staging, and prior drug exposure. DNA mutation and RNA expression profiles were acquired for each patient tumor sample where material was available.

To minimize loss of clonal plasma cell populations over time and enable clinical implementation, we developed a rapid screening platform to measure ex vivo sensitivity of the MMDP in CD138+ MM primary cells with 24 h drug exposure. Our in vitro and ex vivo data were highly concordant, detecting dose-dependent drug sensitivity for 93% of the MMDP drugs.

We acknowledge that primary MM cells would likely prove generally less sensitive in the presence of adherent marrow stroma but believe that the overall validating conclusion, *e.g.*, with respect to venetoclax, proteasome inhibition, selinexor, and IDH mutation, compellingly highlights the utility and knowledge gained from the assay even without stromal elements. Indeed the system was designed with clinical utility in mind; with short turnaround time, this approach is appealing to routine clinical practice whereas standardizing a stromal microenvironment for quality-controlled, reproducible clinical utility would be challenging, if not impossible. Our current screening system was also not able to incorporate immune therapies, such as monoclonal antibodies and checkpoint inhibitors, which have shown promising anti-MM activity^18,51^. While the bone marrow microenvironment was not captured in our assay, patterns of sensitivity, as seen for PIs, dexamethasone, venetoclax, and selinexor, validated the potential clinical utility even without artificial support systems. However, drugs requiring longer incubation times, including notably the IMiDs, were generally not interpretable at 24 h. A parallel 72 h or 96 h ex vivo assay was recently deployed for these drugs; however, currently an insufficient number of samples studied under these conditions were available for inclusion and will be reported later. By contrasting the MMDP sensitivities in HMCLs *versus* NHLCLs, we noted that 43% of the drugs had discernible differences in sensitivity between MM and lymphoma but the highest MM specificity was observed for signal transduction KIs.

By linking our ex vivo drug sensitivity data to clinical, cytogenetic, and mutational profiles, our study provided further insights into contextual sensitivities that may be revealing of MM vulnerabilities and could point to novel opportunities for future drug development. For example, in the case of *IRF4* mutations, known to associate with a favorable overall survival^50^, increased sensitivity to MAPK and ALK inhibitors was prominent. We also found that targeting cell cycle with CDK and DNA synthesis inhibitors may be advantageous for samples harboring 17p deletions.

Furthermore, our deeper look into venetoclax responses indicated an increased sensitivity in MM patients classified as newly diagnosed, standard risk, with low plasma cell S-Phase, harboring t(11;14), and lacking Gain(1q) or t(4;14). Ex vivo functional testing as an alternative approach to predict clinical response to venetoclax had been previously reported^52^. Our data added depth to previous reports of *BCL2* family profiles as a paramount predictor of response to venetoclax^53–55^, supporting *BCL2* expression, *BCL2*/*MCL1*, and *BCL2*/*BCL2L1* transcriptomic ratios as crucial determinants of ex vivo response. Our results additionally highlighted less common non-t(11;14) tumors with sensitivity, which was confirmed in at least one patient through clinical study, strengthening the rationale for an evidence-based therapeutic approach integrating ex vivo and genomic primary sample profiling to guide therapeutic selection for MM.

Our platform also revealed contextual sensitivities for the exportin 1 inhibitor selinexor. Although the drug has shown notable activity, including in penta-refractory MM cases, its reported adverse effect profile may be clinically limiting^56–60^. Therefore, “direct-to-drug” strategies for patient selection may avoid unnecessary toxicity in patients unlikely to benefit and potentially retain sensitivity at lower doses in highly sensitive responders. Increased sensitivity was identified in samples with specific biomarkers of poor prognosis, supporting the use of selinexor in heavily pretreated MM patients.

Hierarchical clustering highlighted six patient subpopulations with distinct drug sensitivity patterns, one of which was enriched in relapsed samples with genetic aberrations reflective of poor prognosis, including t(4;14) and Gain(1q). These samples had the highest selinexor sensitivity and the lowest venetoclax sensitivity. This patient subpopulation was also concomitantly sensitive to DNA synthesis inhibitors and signal transduction inhibitors targeting various kinases including MAPK. These results corroborate a previous report, who postulated that these susceptibilities could point to escape routes for MM survival in refractory disease^26^.

We have initiated a chemogenomic resource capturing nascent associations between clinical and genomic MM profiles and their susceptibility to drugs with clinical utility in MM. As the resource grows, we anticipate that the mosaic of drug sensitivity and associated clinical phenotypes will fuel a personalized medicine engine, which will produce novel targets, novel combinations, novel use cases, and a valuable database for drug developers to access for potential development of clinical biomarkers of response. This work also lays the foundation for future clinical trials exploring this approach.

## Supplementary information

Supplemental Table T1

Supplemental Table T2

Supplemental Table T3

Supplemental Table T4

Supplemental Table T5

Supplemental Table T6

Supplemental Table T7

Supplemental Table T8

Supplemental Table T9

Supplemental Table T10
